# Vesicle Induced Receptor Sequestration: Mechanisms behind Extracellular Vesicle‐Based Protein Signaling

**DOI:** 10.1002/advs.202200201

**Published:** 2022-03-01

**Authors:** Oskar Staufer, Jochen Estebano Hernandez Bücher, Julius Fichtler, Martin Schröter, Ilia Platzman, Joachim P. Spatz

**Affiliations:** ^1^ Department for Cellular Biophysics Max Planck Institute for Medical Research Jahnstraße 29 Heidelberg D‐69120 Germany; ^2^ Institute for Molecular Systems Engineering (IMSE) Heidelberg University Im Neuenheimer Feld 225 Heidelberg D‐69120 Germany; ^3^ Max Planck‐Bristol Center for Minimal Biology University of Bristol 1 Tankard's Close Bristol BS8 1TD UK; ^4^ Max Planck School Matter to Life Jahnstraße 29 Heidelberg D‐69120 Germany; ^5^ Biophysical Engineering of Life Group Max Planck Institute for Medical Research Jahnstraße 29 Heidelberg D‐69120 Germany

**Keywords:** bottom‐up synthetic biology, CD95, ectosomes, Fas, FasL, immunological synapse, receptor multimerization

## Abstract

Extracellular vesicles (EVs) are fundamental for proper physiological functioning of multicellular organisms. By shuttling nucleic acids and proteins between cells, EVs regulate a plethora of cellular processes, especially those involved in immune signalling. However, the mechanistic understanding concerning the biophysical principles underlying EV‐based communication is still incomplete. Towards holistic understanding, particular mechanisms explaining why and when cells apply EV‐based communication and how protein‐based signalling is promoted by EV surfaces are sought. Here, the authors study vesicle‐induced receptor sequestration (VIRS) as a universal mechanism augmenting the signalling potency of proteins presented on EV‐membranes. By bottom‐up reconstitution of synthetic EVs, the authors show that immobilization of the receptor ligands FasL and RANK on EV‐like vesicles, increases their signalling potential by more than 100‐fold compared to their soluble forms. Moreover, the authors perform diffusion simulations within immunological synapses to compare receptor activation between soluble and EV‐presented proteins. By this the authors propose vesicle‐triggered local clustering of membrane receptors as the principle structural mechanism underlying EV‐based protein presentation. The authors conclude that EVs act as extracellular templates promoting the local aggregation of membrane receptors at the EV contact site, thereby fostering inter‐protein interactions. The results uncover a potentially universal mechanism explaining the unique structural profit of EV‐based intercellular signalling.

## Graphical abstract

A molecular signaling mechanism for proteins displayed on extracellular vesicles (EVs) is presented. The authors find that EVs act as concentration devices that restrict diffusion of receptor‐ligand‐complexes to a small area on target cell membranes, boosting receptor multimerization and signaling. The findings suggest that cells apply EVs to presented proteins in order to increase their signaling potency compared to soluble proteins.

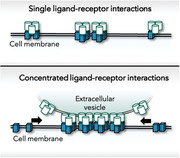

## 1 Introduction

EVs have been pinpointed as pivotal paracrine signalling factors which convey a diverse set of bioactive cargos (e.g., proteins, nucleic acids, and sugars) between cells.^[^ ^1^ ^]^ The fundamental importance of EVs in cell biology is highlighted by the fact, that almost all cell types across the whole phylogenetic tree apply EVs for intercellular communication. Based on tremendous research efforts within the last decades, considerable insights into their biogenesis and enrolment in different intercellular signalling pathways has been gained.^[^ ^2^ ^]^ However, detailed knowledge on their biophysical signalling principles and the mechanism by which they trigger such diverse biological responses is still lacking.^[^ ^3^ ^]^ In this regard, one missing piece of the puzzle is a mechanistic explanation that reveals why cells employ EV‐based communication.^[^ ^4^ ^]^ Of note, concerning the transfer of nucleic acids between cells, EVs offer some unique advantages like cargo protection inside the EV lumen and organotropism.^[^ ^5^ ^]^ However, there is a lack of mechanistic models explaining why proteins like the immunological regulators PD‐L1,^[^ ^6^ ^]^ TNF,^[^ ^7^ ^]^ FasL,^[^ ^8^ ^]^ NKG2D,^[^ ^9^ ^]^ or MHCs^[^ ^10^ ^]^ are presented on EVs. This is of special interest, as most of these proteins are also secreted in a soluble form by cells. Therefore, the following questions arise: why do cells apply vesicular forms of protein ligands and not soluble forms? And how can EVs promote protein‐based signalling?

**Figure 1:**
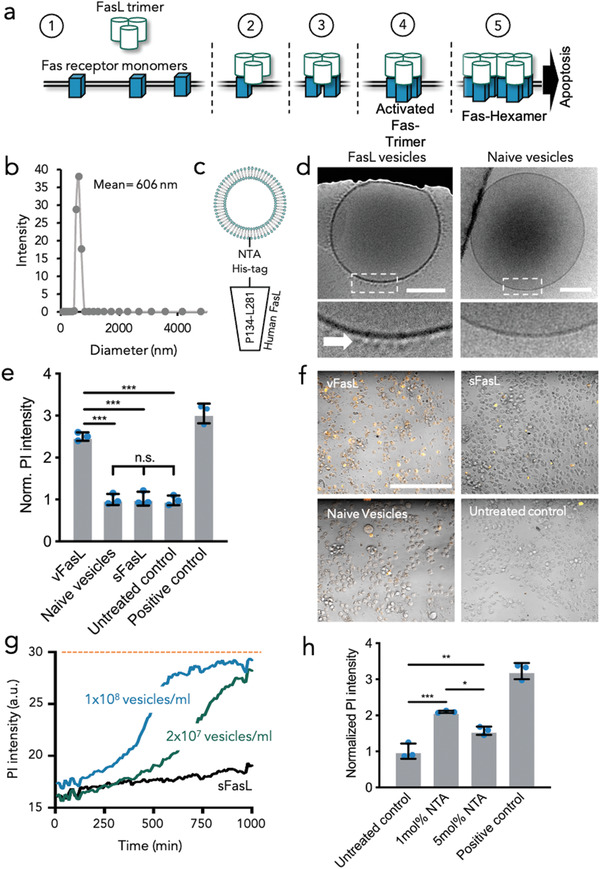
Bottom‐up assembly of vFasL vesicles. a) Schematic illustration of FasL mediated Fas hexamerization in apoptotic signaling. b) Dynamic light scattering size‐analysis of vesicles produced by charge‐mediated assembly within water‐in‐oil droplets. c) Schematic illustration of vesicle functionalization with recombinant human FasL (P134‐L281) fused to an N‐terminal His‐tag. d) Representative cryoTEM micrograph of a FasL decorated vesicle (left) and naive vesicles (right). Magnified insets are shown from the dotted regions above. Scale bars are 200 nm and the arrow points towards immobilized vFasL. e) Quantification of PI staining intensity in Jurkat cell cultures treated with vFasL and sFasL at 28 ng mL^−1^ or naïve vesicles after 24 h of incubation. Tween‐20 treated cultures as positive controls. Results are shown as mean ± SD from three biological triplicates. f) Representative confocal microscopy images of Jurkat cell cultures stained with anti‐caspase (cleaved Asp391) (orange). Cells were either left untreated (negative control) or treated with vFasL vesicles, sFasL, or naïve vesicles for 24 h. Scale bar is 400 µm. g) Time‐resolved PI staining analysis of Jurkat cultures treated with 28 ng mL^−1^ sFasL (black), 1 × 10^8^ mL^−1^ vFasL vesicles (blue), and 2 × 10^7^ mL^−1^ vFasL vesicles (green) corresponding to 28 and 5.6 ng mL^−1^ FasL, respectively. Orange line indicates PI staining intensity of Tween20‐treated positive control cultures. Line profiles are average of three technical replicates. h) Quantification of PI staining intensity in Jurkat cell cultures treated with 28 ng mL^−1^ vFasL on vesicles harboring 1 mol% and 5 mol% DGS‐NTA(Ni^2+^) after 24 h of incubation. Results are shown as mean ± SD from *n* = 3 biological triplicates. *p < 0.05, **p < 0.005, ***p < 0.0005, and n.s. = not significant with one‐way ANOVA analysis and Bonferroni post‐hoc testing.

Some of the proteins found on EV membranes are associated to EV maturation and shedding or mediate targeting and cellular uptake.^[^ ^2^ ^]^ However, a substantial fraction of the EV membrane proteins exhibit specific effector functions, which are for instance fundamental for neurodevelopmental^[^ ^11^ ^]^ and immunological signalling.^[^ ^10^, ^12^ ^]^ Particularly, cytotoxic T‐ and NK‐cells directly release EVs decorated with Fas ligand (FasL, CD95L, CD178) into cytotoxic immunological synapses, for instance during activation‐induced cell death.^[^ ^13^ ^]^ The Fas receptor is a member of the prevalent TNF‐receptor superfamily and therefore a prime example to study EV‐based protein presentation. Vesicular FasL (vFasL), presented on EV surfaces, is essential for cellular cytotoxicity, since by binding to the target cell receptors, it induces caspase‐mediated apoptosis.^[^ ^14^ ^]^ Importantly, apoptosis is initiated by FasL‐mediated Fas‐receptor trimerization on the target cell membrane, which activates and subsequently hexamerizes the Fas receptor leading to the formation of a death inducing signalling complex (DISC) (**Figure** **1**a).^[^ ^15^ ^]^ However, cytotoxic cells can also shed FasL in a non‐vesicular but soluble form (sFasL). This is achieved by extracellular matrix‐metalloprotease cleavage of transmembrane FasL on the extracellular stalk domain.^[^ ^16^ ^]^ Several previous reports show that in contrast to vFasL, sFasL is not a potent inducer of Fas‐mediated apoptosis.^[^ ^17^ ^]^ Importantly, this effect is not based on differences in receptor avidity or multivalent interactions between sFasL and vFasL^[^ ^18^ ^]^ but depends on FasL lateral mobility.^[^ ^19^ ^]^ It has been proposed that the differences in apoptotic‐efficiency between membrane‐bound FasL and sFasL relays in their ability to induced Fas hexamerization and higher‐order oligomerization.^[^ ^15a^ ^]^ In this case, membrane‐bound FasL induces higher hexamerization rates, leading to more efficient DISC formation and higher apoptosis rates. However, it remains a long‐standing question whether and how the signalling potency of FasL (and other proteins) is affected by its immobilization and presentation on EV surfaces. Resolving this issue, could not only impact on our understanding of Fas‐signalling associated immunological processes but reveal a universal mechanism underlying the “template” function of EVs for protein presentation.

In this study, we aimed to investigate the fundamental principles that potentiate the signalling function of protein ligands presented on EV membranes. Towards this, we chose FasL‐signalling as a prime example to study this phenomenon due to the following three major reasons: 1) the molecular signalling mechanisms, underlaying Fas activation are well studied;^[^ ^15a^ ^]^ 2) there is direct experimental evidence for the involvement of EVs in the Fas‐FasL signalling axis and there is experimental proof for a site‐directed release of EVs from one cell towards another cell as well as;^[^ ^13b^ ^]^ 3) both types, sFasL and vFasl, have been studied and found to display differing cytotoxicity.^[^ ^17^, ^20^ ^]^ To perform a quantitative and systematic assessment of this EV‐signalling axis, we apply techniques and principles inspired from bottom‐up synthetic biology in order to assemble lipid vesicles mimicking natural protein presenting EVs.^[^ ^21^ ^]^ The fundamental advantage of bottom‐up synthetic biology compared to classical top‐down engineering of natural systems lies in the controlled and defined assembly of such synthetic constructs.^[^ ^22^ ^]^ This allows us to break‐down the complexity of natural systems and quantitatively engineer individual components. We combine this bottom‐up approach with the modelling of ligand and receptor diffusion on vesicle membranes. This combination provided a quantitative and mechanistic understanding of vesicle‐mediated receptor‐ligand interactions. Based on our results, we propose that vesicle induced receptor sequestration (VIRS) is a universal mechanism underlying EV‐based intercellular signalling and responsible for EV‐mediated potentiation of the signalling activity of receptor ligands.

## 2 Results

### 2.1 Bottom‐Up Assembly and Characterization of FasL Biofunctionalized Vesicles

We previously reported on microfluidic and bulk methodologies to produce large amounts of highly defined liposomal vesicles in diverse size ranges, which we applied for bottom‐up assembly of synthetic cells, organelles, and EVs.^[^ ^21^, ^23^ ^]^ These charge‐mediated vesicle assembly technologies allow for high‐throughput and sequential production of molecularly defined unilamellar vesicles. These synthetic vesicles can be decorated with transmembrane and peripheral proteins to tune their interactions with cells (see Note S1, Supporting Information).^[^ ^24^ ^]^ Comparable vesicles have been extensively used in bottom‐up synthetic biology to uncover fundamental dynamics in cell biology and intercellular signalling.^[^ ^22^, ^24^, ^25^ ^]^ Due to their structural and molecular similarity to natural EVs, we reasoned that such fully‐synthetic EVs (fsEVs) could serve as powerful platforms to explore EV‐based protein presentation.

We first produced fluorescently labelled fsEVs from phosphatidylglycerol (EggPG) and phosphatidylcholine (EggPC) lipids to mimic the high PC content as well as negative charge of natural EVs. Biorthogonal functionalization of the fsEVs was achieved with DGS‐NTA(Ni^2+^) lipids for protein coupling and Atto488 or Rhodamine B conjugated phosphatidylethanolamine lipids for microscopy observation. We measured size and zeta‐potential of fsEV by dynamic light scattering (DLS) and found a mean diameter of 606 nm, which is within the size spectrum of natural EVs (Figure 1b), and a zeta‐potential of −21.2 ± 0.8 mV (*n* = 3 technical replicates). Recombinant ectodomains of human FasL (Pro134–Leu281) were immobilized by an N‐terminal 8xHistidine‐tag on the vesicles via 1mol% DGS‐NTA(Ni^2+^) conjugated lipids (Figure 1c). The immobilization of FasL on the vesicle membrane was verified by cryogenic transmission electron microscopy (cryoTEM) (Figure 1d). To assess the functionality of the immobilized ligands, we analyzed the pro‐apoptotic potential of these vesicles by incubating them for 24 h with Fas‐expressing Jurkat T‐lymphocytes. Cytotoxicity was subsequently assessed by propidium iodide (PI) staining of dead cells and evaluated by live‐cell fluorescence microscopy and plate‐reader based bulk fluorescence intensity measurements of the cultures (see Experimental section). Furthermore, PI dead cell staining was correlated with Annexin V staining assessed by flow cytometry to quantify early apoptotic events and verification of the PI bulk staining (see Figure S1, Supporting Information). Importantly, when quantifying the PI intensity between cells treated with vFasL and cells treated with the same concentration of sFasL, we found that only vFasL exerted a considerable pro‐apoptotic effect (Figure 1e). Note, for both conditions (sFasL and vFasL), the very same recombinant FasL, from exactly the same purification lot, was used. Therefore, differences in the biochemical characteristics of sFasL and vFasL could be ruled out. Moreover, we found by time‐laps fluorescence microscopy, that the cells rapidly formed apoptotic morphologies upon vesicle‐binding to their membrane (Video S1, Supporting Information). We observed a stable attachment of the vesicles to cells after contact formation. Even after formation of apoptotic blebs, detachment of the vesicles after cell death was only rarely observed during the experimental time frame of 12 h. Notably, the considerable pro‐apoptotic effect of vFasL in comparison to sFasL could be observed also when targeting several different cell lines like K562 human leukemia cells and BJ human dermal fibroblasts (sees Figure S2, Supporting Information).

To verify that vFasL induced “correct” caspase‐mediated apoptosis and does not trigger any necrotic effects, we further confirmed Asp391 caspase‐8 cleavage by immunostaining and flow cytometry quantification (Figure 1f and Figure S3, Supporting Information). Moreover, we performed antibody staining against Fas‐associated protein with death domain (FADD) to observe accumulation of FADD at the vesicles contact side, indicative for DISC formation (see Figure S4, Supporting Information). In order to compare the pro‐apoptotic potential of vFasL to sFasL, we performed dose‐response experiments for vFasL and sFasL. Bulk PI staining analysis revealed, that over 1000‐fold more sFasL is needed to reach equivalent vFasL apoptosis rates, highlighting the effect of ligand immobilization of vesicle membranes (see Figure S5, Supporting Information). Taken together, this illustrates that simple immobilization of sFasL on a vesicle membrane transforms it into a potent apoptosis inducer and highlights the importance of vesicle‐based protein presentation on intercellular signalling.

In a next step, we analyzed vFasL signalling on a more quantitative level by testing the effects of varying vFasL densities on the vesicles, keeping the overall FasL concentration constant by adjusting the total number of vesicles added. In addition, we assessed the impact of varying vesicle concentrations incubated with the cells. By time‐resolved PI intensity bulk measurements of these cultures, we found that the killing rate is dependent on vesicle concentration, where 1 × 10^8 ^vesicles/mL reached positive control (tween‐20 detergent treatment) levels approximately twice as fast as 1 × 10^7 ^vesicles/mL (Figure 1g). In order to analyze the effect of vFasL membrane density on the pro‐apoptotic potential, we produced vesicles harboring 5mol% DGS‐NTA(Ni^2+^) and compared these to the 1 mol% DGS‐NTA(Ni^2+^) vesicles. In order to keep the final vFasL concentration incubated with Jurkat cells constant, we applied either 1 × 10^8 ^mL^–1^ vesicles with 1mol% DGS‐NTA(Ni^2+^) or 2 × 10^7^ vesicles with 5 mol% DGS‐NTA(Ni^2+^). Of note, the correlation between DGS‐NTA(Ni^2+^) concentration in the vesicle membrane and recombinant ligand density was verified by labelling the vesicles with gold‐nanoparticle conjugated antibodies and subsequent transmission electron microscopy imaging (see Figure S6, Supporting Information). We found that the vesicles with higher FasL density displayed reduced killing‐efficiency (Figure 1h). This demonstrates that FasL density on the vesicle membrane is not a limiting factor for vFasL‐triggered apoptosis but vesicle concentration majorly impacts on the killing dynamics.

Taken together, these experiments demonstrate that simple immobilization of a protein ligand on a vesicle membrane can “awake” its signalling functionality. As the very same FasL samples were used for preparation of sFasL and vFasL, the mechanism underlying this effect is most likely not a biochemical or structural modification of the ligand but likely associated to a fundamental biophysical phenomenon mediated by the vesicles. In this context, effects associated to the diffusion of receptors and ligands in the membrane as well as their multimerization dynamics are likely to provide insight into the vesicle‐promoted signalling. Towards resolving these aspects, diffusion simulations can be applied for the visualization of emerged properties arising from time‐dependent dynamics in receptor‐ligand interactions.^[^ ^26^ ^]^

### 2.2 Diffusion Simulation of vFasL Signaling

**Figure 2:**
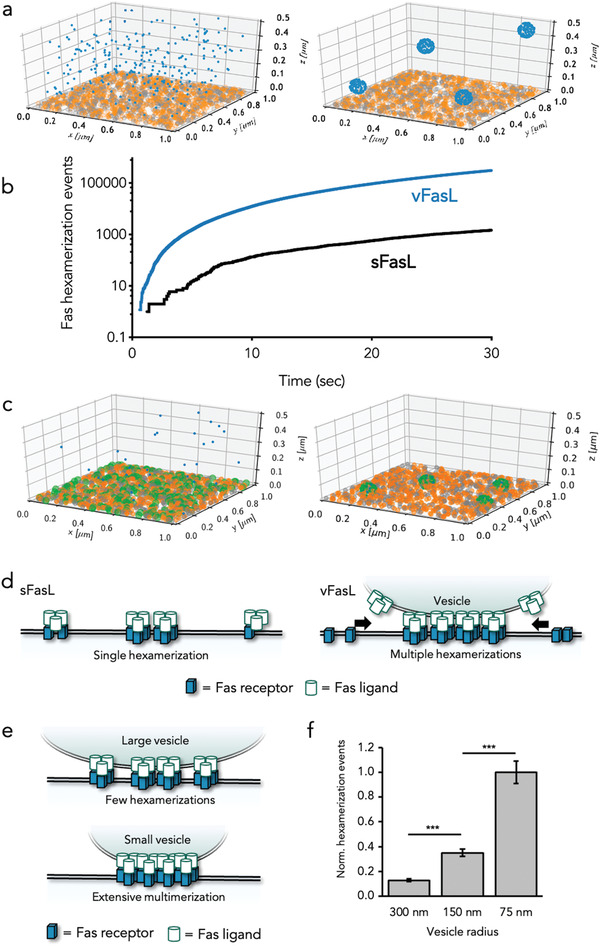
Diffusion simulations of sFasL and vFasL in an immunological synapse. a) Exemplary illustrations of FasL released into the simulated immunological synapse as sFasL (left) and vFasL (right). FasL, monomeric Fas, and dimeric Fas are shown in blue, orange, and grey, respectively. b) Cumulative Fas hexamerization events for sFasL and vFasL simulation over a time period of 30 s. c) Exemplary illustrations of FasL‐hexamerized Fas distribution in synapses simulated with sFasL (left) and vFasL (right) after 30 s. FasL, monomeric Fas, dimeric Fas, and hexameric Fas is shown in blue, orange, grey, and green, respectively. d) Schematic illustration of vesicle‐induced receptor sequestration mediating increased inter‐receptor interactions at the vesicle attachment site. e) Schematic illustration of vesicle size effects in the VIRS concept. f) Comparison of cumulative hexamerization events observed in diffusion simulations over a time period of 30 s induced by vFasL vesicles with varying diameter but constant FasL density. Results are shown as mean ± SD from *n* = 32 individual simulations. ***p < 0.0005 and n.s. = not significant with one‐way ANOVA analysis and Bonferroni post‐hoc testing.

In order to elucidate the ligand‐receptor interactions during vesicle‐based presentation, we combined our bottom‐up synthetic biology approach with diffusion simulations. Specifically, we aimed to probe Fas‐FasL binding and multimerization dynamics by vFasL and sFasL on a target cell membrane. Towards this, we implemented a “diffusion space” with 2 µm × 2 µm × 2 µm, corresponding approximately to the size of an immunological synapse deduced from previous electron microscopy observations.^[^ ^27^ ^]^ On the lower surface of this space, the target cell membrane harboring Fas receptors is modelled. Into the cleft, a pre‐defined amount of trimeric sFasL molecules or vFasL vesicles are “released” and allowed to diffuse (**Figure** **2**a). Diffusion of sFasL was simulated based on a Brownian random walk model of massless point particles with short‐range Yukawa interaction potentials.^[^ ^28^ ^]^ For diffusion modelling of membrane bound Fas and Fas‐FasL multimerized complexes, a diffusion model based on the Saffman–Delbrück theory was applied to calculate the diffusion coefficients of membrane proteins (see Note S2 for details on the simulation as well as Table S1 for simulation parameters, Supporting Information). As Fas‐FasL binding and Fas‐multimerization are the most critical events regulating Fas‐signalling,^[^ ^15a^ ^]^ the number of sFasL or vFasL bound Fas receptors, the amount of trimerized FasL as well as events of Fas‐hexamerization were counted by the simulation over time (time interval 1 ms). The model was based on three major assumptions concerning the biological processes: first, it is assumed that the number of Fas hexamerizations correlates with the apoptotic potency of FasL, as supported by previous analysis.^[^ ^29^ ^]^ Second, once FasL has bound to Fas, the unbinding kinetic was assumed to be neglectable in the relevant time frame. This is supported by previous studies that found trimeric Fas‐FasL dissociation constant of 4.1 × 10^–4^s^–1^.^[^ ^30^ ^]^ Third, it was assumed that sFasL and vFasL predominantly exist in a trimeric form and that these trimers induce the trimerization of Fas. This is supported by previous literature reports and our own measurements of the sFasL hydrodynamic radius (see Figure S7, Supporting Information).^[^ ^15c^ ^]^

The main readout of our simulations is the cumulative number of Fas hexamerization events over time. A direct comparison of sFasL and vFasL diffusing in the simulated immunological synapse, revealed that the cumulative increase in hexamerization events over a time frame of 30 s is significantly slower for sFasL (Figure 2b). This was the case, as once a FasL‐trimer molecule trimerized Fas, this activated complex would randomly diffuse over a large membrane area and continuously interact at the same rate and probability with other activated Fas trimers over time. In the case of vFasL however, the cumulative hexamerization events were approximately over 100 times higher, once a vesicle was bound to the target cell membrane. This is in good agreement with our experimental results were sFasL showed almost no killing compared to vFasL. Of note, our simulation also accounted for the slower diffusion of vFasL vesicles compared to the much smaller sFasL molecules. Despite this fact, vFasL still displays faster Fas hexamerization on the target membrane (see initial offset between sFasL and vFasL in Figure 2b). Based on our simulations, we could observe that this effect was not due to a significantly higher FasL concentration in close proximity to the target cell membrane. In fact, the possibility that such a local concentration effect increases sFasL apoptotic signalling, has also been experimentally ruled out by Kallenberger et al.^[^ ^15b^ ^]^ The most striking difference between the two FasL presentation “modes” was that vFasL generated a locally concentrated pool of activated, FasL‐trimerized Fas below the vesicles binding site (Figure 2c). In contrast, sFasL‐trimerized Fas randomly diffused over the whole target cell membrane. Therefore, the EV contact site acts as an extracellular template which successively sequestrates more and more Fas receptors and promotes interactions between them by restricting their lateral diffusion to a condensed region on the target cell membrane (Figure 2d). Following the results of these simulations, vFasL harboring EV act by a vesicle‐induced receptor sequestration (VIRS) mechanism.

A further hypothesis arising from our simulations, which supports the view of a VIRS effect in EV‐signalling, were that hexamerization events are to some extend inverse proportional to the vesicle radius. In this case, the vesicle size limits the “adhesion” area of the vesicle with the target cell membrane. According to the VIRS model, smaller vesicles with constant FasL number are expected to induce a higher local concentration of Fas receptors compared to larger vesicles (Figure 2e). Accordingly, when performing our simulations with vesicles of varying radius but constant FasL density, we found that the total hexamerization events after 30 s were significantly higher for smaller vesicles (Figure 2f).

### 2.3 Experimental Confirmation of the VIRS Mechanism by Bottom‐Up Assembly of Synthetic EVs

In a next step, we aimed to experimentally validate the VIRS effect observed in our simulations by imaging Fas membrane localization in cells incubated with FasL‐decorated vesicles. We chose adherent Hela cells that were transfected with a Fas‐GFP fusion constructs (see Experimental Section). Compared to approximately spherical suspension Jurkat cells, Hela cells grown as adherent cultures, display larger planar membrane areas that are suitable to study of membrane protein diffusion (e.g., by FRAP analysis). This enables more accurate quantification of receptor clustering and diffusion. Moreover, Hela cells display higher transfection efficiencies compared to Jurkat cells, facilitating the search for Fas‐GFP positive cells in direct contact with a vFasL vesicle. Hela cells were transfected for 24 h, incubated for 2 h with vFasL in order to capture initial events of Fas hexamerization and subsequently imaged by fluorescence confocal microscopy at the sites of vesicle–cell membrane interactions. Fas localization was imaged by the GFP tag.

**Figure 3:**
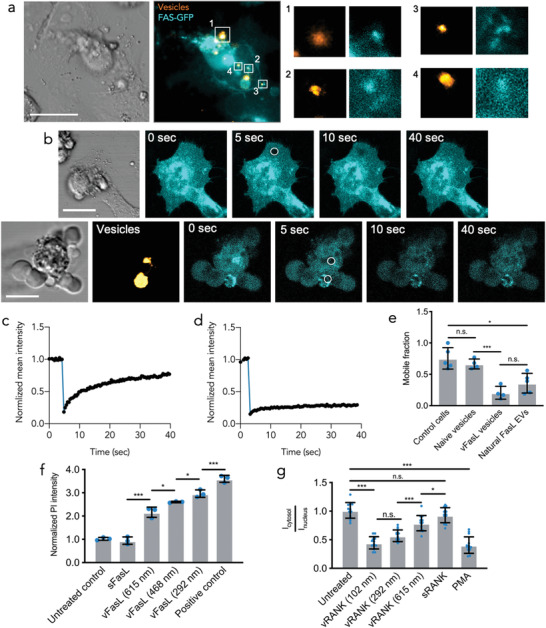
Receptors sequestration by vesicle‐immobilized ligands. a) Bright field (grey) and maximal z‐projections from confocal microscopy stacks of Hela cells expressing Fas‐GFP fusion proteins (cyan) incubated with vFasL vesicles (orange). Right side single channel images are exemplary single plane magnifications from boxed regions indicated left. Scale bar is 20 µm. b) Representative bright field (grey) and confocal microscopy images of Hela cells expressing Fas‐GFP fusion proteins (cyan). Top row shows control cells cultured without vFasL vesicles and bottom row shows apoptotic cells incubated with vFasL vesicles (orange). Encircled areas indicated bleaching spots used for FRAP analysis. Scale bars are 20 µm. c,d) Exemplary normalized fluorescence intensity recovery time profiles of cell membrane regions in control cells (c) and cell membrane regions in contact with a vFasL vesicle (d) from imaging experiments shown in (b). e) Quantification of Fas‐GFP mobile fractions based on the FRAP analysis. Mobile fractions of Fas‐GFP are shown for untreated control cells as well as at the attachment site of naive vesicles, synthetic vFasL vesicles, and natural FasL‐enriched EVs. Results are shown as mean ± SD, pooled from two independent experiments in each condition and every data point indicates mobile fraction analysis from a single cell‐vesicle contact site with *n* > 4. **p < 0.005 and n.s. = not significant with one‐way ANOVA analysis and Bonferroni post‐hoc testing. f) Quantification of bulk PI staining intensity in Jurkat cell cultures treated with vFasL vesicles of varying diameter. Results are shown as mean ± SD from *n* = 3 biological triplicates. *p < 0.05, ***p < 0.0005, and n.s. = not significant with one‐way ANOVA analysis and Bonferroni post‐hoc testing. g) Quantification of the ratio of anti‐p(202/204)ERK1/2 staining intensity in the cytosol and nucleus of MC3T3 cell cultures treated with sRANK and vRANK presented on vesicles of varying diameter, basal levels in untreated control cultures, and PMA treated cultures as positive control. Results are shown as mean ± SD from *n* = 14 individual cells. *p < 0.05, ***p < 0.0005, and n.s. = not significant with one‐way ANOVA analysis and Bonferroni post‐hoc testing.

We found punctuated spots of increased Fas‐GFP intensity in the plasma membrane where vFasL vesicles were attached (**Figure** **3**a). This indicates that vesicle‐immobilized ligands can recruit receptors to the vesicle attachment side. In order to analyze if vFasL not only recruits and promotes clustering of Fas receptors but also sequesters them, we performed fluorescence recovery after photobleaching (FRAP) experiments (Figure 3b). This allowed us to study the free lateral diffusion of GFP‐Fas receptors in the cell membrane as well as any restricted diffusion at the vesicle contact sites. For cells that were not incubated with vFasL vesicles, we found a Fas‐GFP diffusion coefficient of 2.53 ± 1.29 µm^2^ s^–1^ (*n* = 4 individual bleaching spots) in the cell membrane. In contrast, Fas‐GFP diffusion coefficient was significantly reduced at the sites of vFasL‐vesicle contact (0.30 ± 0.18 µm^2^ s^–1^, *n* = 4 individual bleaching spots) (Figure 3c,d). Most importantly, our FRAP experiments revealed that the mobile fraction of Fas‐GFP receptors at the vesicle contact site was only 0.21 ± 0.1 (*n* = 4 individual bleaching spots) and therefore significantly reduced compared to “free” Fas‐GFP (0.69 ± 0.11, *n* = 4 individual bleaching spots) (Figure 3e). Importantly, this effect could only be observed for FasL‐decorated vesicles and not for naive vesicles, ruling out perturbations of Fas‐diffusion by non‐specific interaction with the lipid vesicles. Furthermore, this observation was also made when testing natural FasL‐enriched EVs (see Figure 3e), which underscores that the VIRS model does not only apply for synthetic vesicles systems but can also be translated towards natural EV particles. This demonstrates that the majority of Fas receptors at the vesicle contact site are laterally non‐mobile and sequestered by the vesicles.

In order to verify this size‐dependent prediction from our diffusion simulations experimentally and to further validate the VIRS model, we bottom‐up assembled vesicles of varying radii but constant vFasL densities and tested their apoptotic potential on Jurkat cells. In line with our simulation results, we found that when varying vesicle size between 615 and 292 nm, the smaller fsEVs induced increased apoptotic rates (Figure 3f). We further applied a mechanical vesicle extrusion technique to assemble vesicles in a size range <200 nm in order to verify these results. Although vesicle extrusion does not provide the same degree of control compared to our droplet‐based assembly, it allows for much smaller vesicle sizes. We found that the killing efficiency is further increased with vesicles of 189, 122, and 86 nm (see Figure S8, Supporting Information). These experimental observations not only support our simulation results but also underline the templating effect of vesicles for ligand presentation as well as the importance of their physical properties (e.g., size of the vesicle).

Next, we aimed to assess if VIRS is specific for FasL signalling or also applies to other protein ligands and therefore might represent a potentially universal mechanism for EV‐based protein presentation. We therefore investigated the role of VIRS in receptor activator of NF‐*κ*B (RANK) decorated vesicles. RANK‐signalling is essential for the homeostasis of osteoblast‐osteoclast balance in bone resorption and mediated by a MAPK/ERK signalling pathway.^[^ ^31^ ^]^ Moreover, RANK revers signalling has been shown to relay on EV‐based shuttling of RANK presenting vesicles.^[^ ^32^ ^]^ We therefore produced vesicles of varying size decorated with recombinant C‐terminal Histidine‐tagged ectodomains of mouse RANK (Val31‐Asp214) immobilized via NTA(Ni^2+^) lipids. Respective vesicles were incubated with mouse MC3T3 osteoblast cells and ERK1/2 signalling was quantified by antibody staining for ERK1/2 (p202/204) after 24 h of incubation. In this assessment, ERK1/2 phosphorylation is a measure for the activation of RANK reverse signalling via the MAP‐kinase pathway.^[^ ^33^ ^]^ Quantification was performed by assessing nuclear translocation of phosphorylated ER1/2 with 12‐myristate 13‐acetate (PMA) as positive control for MAP‐kinase signalling (see methods). Remarkably, also in this setting we found that ERK1/2 phosphorylation correlates with vesicles size, where smaller vesicles induced higher ERK1/2 phosphorylation rates (Figure 3g and Figure S9, Supporting Information). Moreover, this analysis revealed that soluble RANK (sRANK), at the applied concentration of 28 ng mL^–1^, is not able to induce ERK1/2 (p202/204) phosphorylation above untreated control levels, while vesicular RANK (vRANK) induced a prominent ERK1/2 phosphorylation. Taken together, this suggests that VIRS does not only apply to FasL‐based vesicular signalling but that this mechanism might underlie diverse EV‐based signalling routs.

## 3 Conclusion

In this study, we describe a potentially universal mechanism underlying EV‐based protein presentation. Our experimental approach deviates from convectional top‐down engineering of EV‐systems and applies a strategy that is based on a bottom‐up assembly of EV‐like vesicles. As this approach allows for a precise control over the vesicle characteristics, we were able to specifically modulate key biophysical characteristics of the vesicles like size or ligand density and to elucidate EV‐signalling in quantitative manner. Most importantly, we were able to show that simple immobilization of a receptor ligand on a vesicles membrane dramatically increases its signalling potential. Several previous reports described comparable observation on natural EVs, where soluble ligands showed far inferior or completely adverse effects to their EV‐presented counter parts.^[^ ^7^, ^9^, ^11^, ^34^ ^]^ However, no mechanistic explanation was available yet. In turn, our experimental results revealed that this behavior correlates with the vesicle size. Together with diffusion‐based simulations of receptor‐ligand interactions, we were able to postulate a VIRS mechanism, by which receptors and clusters thereof accumulate at the vesicle‐cell interface. Therefore, EVs can be considered as “concentration devices” for membrane proteins, applied by cells for long‐distance signalling. In consequence, EV templates promote outside‐to‐inside signalling based on self‐organization with unidirectional causality. This allows autonomous self‐organized morphological states reorganize towards external templates.

The simplicity of this mechanism also entails considerable versatility as it potentially applies for all ligand‐receptor interaction requiring multimerization, ligand co‐presentation, intracellular formation of multiprotein complexes, and cross‐interactions thereof. A prime example for this could be TCR signalling: proper antigen presentation by TCR‐based signalling involves clustering of TCR with CD3‐dimers to form functional receptor‐complex signalling transduction units that trigger interprotein cross‐phosphorylation.^[^ ^35^ ^]^ Importantly, TCR is shed from immune cells on EVs.^[^ ^12^, ^36^ ^]^ Potentially, VIRS also applies in this scenario, requiring hetero‐oligomerization of proteins. Fas signalling is a prime example for EV‐signalling, as several studies have demonstrated the directed shedding of vFasL and its physiological relevance. However, the presented study only applies ligands from the TNF‐receptor superfamily (FasL and RANKL), which is a prevalent receptor family with various physiological functions.^[^ ^37^ ^]^ Therefore, the VIRS mechanism remains to be verified for other ligands‐receptor interactions where EV‐based presentation augments signalling potency of the ligand, for example, interleukin presentation of therapeutic exosomes^[^ ^38^ ^]^ or other ligands of receptor tyrosine kinases. Furthermore, it remains to be verified to which extent vesicle internalization after binding and receptor expression levels limits the VIRS effect under in vivo conditions. Moreover, in order to provide a holistic understanding of this effect, future research will need to elucidate the time dynamics underlaying the supramolecular clustering of receptors as well as the life‐time of these structures.

Even beyond EV‐signalling, our results bear direct implications for biomedical application and therapies focused on immunological targeting. FasL has been explored as potential target or effector molecule in various pre‐clinical investigations.^[^ ^39^ ^]^ Uncovering novel and highly effective administration routs and delivery platforms for FasL in the form of synthetic vFasL vesicles, could significantly boost developments in this research line. Finally, our study highlights some of the key advantages of bottom‐up synthetic biology when applied in EV‐focused research. In this regard, reconstitution of artificial life‐like structures, enables uncovering of fundamental principles in non‐equilibrium phenomena and in cellular information processing. Therefore, insights and engineering perspectives hardly achievable by conventional means of top‐down studies in natural systems can be achieved.

## 4 Experimental Section

### Materials

EggPG, EggPC, 18:1 DGS‐NTA(Ni) 1,2‐dioleoyl‐sn‐glycero‐3‐[(N‐(5‐amino‐1‐carboxypentyl)iminodiacetic acid)succinyl] (nickel salt) and extrude sets with 50 nm pore size polycarbonate filter membranes were purchased from Avanti Polar Lipids, USA. 1,2‐Dioleoyl‐sn‐glycero‐3‐phosphoethanolamine labelled with Atto488 was purchased from Sigma Aldrich, Germany. All lipids were stored in chloroform at −20 °C and used without further purification. Hoechst 33 342, Dulbecco's Modified Eagle Medium (DMEM) high Glucose, heat‐inactivated fetal bovine serum, penicillin‐streptomycin (10000 U mL^−1^), L‐Glutamine (200 mm), trypsin‐EDTA (0.05%) with phenol red, anti‐Caspase 8 (Cleaved Asp391) monoclonal antibody clone S.147.8, AlexaFluor555‐conjugated goat anti‐Rabbit IgG (A21429), alpha‐MEM cell culture medium, 4‐(2‐hydroxyethyl)‐1‐piperazineethanesulfonic acid (HEPES), and phosphate buffered saline were purchased from Thermo Fischer Scientific, Germany. 1H,1H,2H,2H‐Perfluoro‐1‐octanol (PFO) de‐emulsifier, Triton X‐100 reduced and propidium iodide were purchased from Sigma Aldrich, Germany. Bovine albumin fraction V (BSA) and paraformaldehyde were purchased from Carl Roth, Germany. Jurkat, MC3T3, K562, BJ cell lines as well as Iscove's Modified Dulbeco's Medium were obtained from ATCC, USA. FC‐40 oil was purchased from io‐li‐tec, Germany. 96‐well plates were purchased from TPP, Switzerland. Purified and Alexa488‐conjugated anti mouse ERK1/2 (pT202/pY204) clone 20A (RUO) was purchased from BD Biosciences. Nunc Lab‐Tek 8‐well chamber were purchased from VWR, Germany. Membrane bound FasL supplied as cell released EVs were obtained from Upstate Cell Signalling solutions (#01‐210). Recombinant human FasL (Pro134‐Leu281) with N‐Met‐8xHis‐tag was purchased from Biolegend, USA. Recombinant mouse RANK (V31‐D214) with C‐terminal 6xHis‐tag and human FADD antibody conjugated to AlexaFluor647 were purchased from Abcam, UK. JetPrime Transfection kit was purchased from PolyPlus Transfection, France. C‐GFPSpark‐tagged Fas pCMV3‐C‐GFPSpark mammalian expression plasmids (MG50027‐ACG) were obtained from Sino Biologicals, China. C‐flat holey carbon‐coated multihole grid was purchased from Protochips, Morrisville, USA.

### Cell Culture

BJ cells were cultured in Dulbecco's Modified Eagle Medium supplemented with 4.5 g L^−1^ glucose, 1% L‐glutamine, 1% penicillin/streptomycin, and 10% fetal bovine serum. MC3T3 cells were cultured in alpha‐MEM medium with ribonucleosides, deoxyribonucleosides, 2 mm L‐glutamine, and 1 mm sodium pyruvate, supplemented with 1% penicillin/streptomycin and 10% fetal bovine serum. Cells were routinely cultured at 37 °C and 5% CO_2_ atmosphere and passaged at ≈80% confluency based on 0.05% trypsin/EDTA treatment. Jurkat and K562 cells were cultured in suspension in Iscove's modified Dulbecco's Medium supplemented with 10% fetal bovine serum. Jurkat and K562 cells were split every other day, at an approximate cell density of 2 × 106 cells/mL, by an approximate 1:4 dilution with fresh cell culture medium.

### Confocal and Bright Field Microscopy

Confocal microscopy was performed with a laser‐scanning microscope LSM 800 (Carl Zeiss AG). Images were acquired with a 20× (Objective Plan‐Apochromat 20x/0.8 M27, Carl Zeiss AG). ImageJ (NIH) was used to analyze the images and adjustments to image brightness and contrast as well as background corrections were always performed on the whole image and special care was taken not to obscure or eliminate any information from the original image. A Leica DMi8 inverted fluorescent microscope equipped with a sCMOS camera and 10× HC PL Fluotar (NA 0.32, PH1) objective was used for bright field and epi‐fluorescence time laps imaging.

### Cryogenic Transmission Electron Microscopy

For cryoTEM analysis, the FasL‐functionalized GUVs were prepared by adding 2.5 µL of the sample solution onto 200 mesh C‐flat holey carbon‐coated multihole grid (Protochips, USA) or ultrafoil gold grids R 2/2 (Quantifoil GmbH, Germany) treated with glow‐discharged. Subsequently, the grids were blotted for ≈4 s and followed by plunge freezing in liquid ethane at 100% humidity using a Vitrobot Mark IV (FEI NanoPort, The Netherlands). Samples were routinely stored in liquid N2. Samples were analyzed with a FEI Tecnai G2 T20 twin transmission electron microscope (FEI NanoPort, The Netherlands) operated at 200 kV. Micrographs were acquired with a FEI Eagle 4k HS, 200 kV CCD camera at an approximate dose of ≈40 electrons/Å^2^.

### Transmission Electron Microscopy

Cryo‐EM samples were prepared by applying 2.5 µL of vesicle solution onto a glow‐discharged 200 mesh C‐flat holey carbon‐coated multihole grid. Subsequently, blotting was performed for 4 s. Plunge‐freezing was performed in liquid ethane using a Vitrobot Mark IV at 100% humidity and grids were stored under liquid nitrogen. The samples were imaged with a FEI Tecnai G2 T20 twin transmission electron microscope operated at 200 kV. A FEI Eagle 4k HS, 200 kV CCD camera was used to record electron micrographs with a total dose of ≈40 electrons/Å^2^.

### Dynamic Light Scattering

Analysis of the hydrodynamic radius of vesicles was performed with a Malvern Zetasizer Nano ZS system. Samples were diluted to a final lipid concentration of 15 µm in PBS filtered with through a 0.22 µm filter. The temperature equilibration time was set to 300 s at 25 °C. Three individual measurements for each sample were performed at a scattering angle of 173° based on the built‐in automatic run‐number selection. The material refractive index was set to 1.4233 and solvent properties were set to *η* = 0.8882, *n* = 1.33, and *ε* = 79.0.

### Vesicle Preparation

Vesicles were produced by formation of droplet‐stabilized GUVs (dsGUVs) by applying a modified shear stress bulk emulsification setup (see Note S1, Supporting Information).^[^ ^40^ ^]^ In brief, a FC‐40 oil phase containing 1.25 mm custom made triblock PEG2500‐PFPE600‐PEG2500 fluorosurfactant^[^ ^41^ ^]^ was mixed in a 2:1 ratio with an aqueous PBS phase containing small unilamellar vesicles (SUVs) of the desired lipid composition. SUVs were produced by mixing lipids dissolved in chloroform stock solutions at the desired lipid ratio in glass a vial. The chloroform was manually evaporated under a gentle nitrogen stream. Subsequently, the lipid film was rehydrated by adding a PBS containing 10 mm MgCl_2_ to obtain a final lipid concentration of 6 mm. The solution was incubated for 15 min and then agitated for 5 min at 1000 rpm. This generates a mixture of liposomal vesicles which were subsequently extruded at least 9 times through a 50 nm pore size filter, resulting in the final SUV solution. For the production of dsGUV, the SUV solution was diluted to a final concentration of 3 mm by adding 10 mm MgCl_2_ containing PBS. This final aqueous phase was added to the perfluorinated oil phase and mechanical emulsification was performed using an Ultra Turrax IKA T10 basic emulsifier for 60 s at ≈26 300 rpm. For preparation of differently sized vesicles (see Figure 3f,g), the emulsification speed of the mechanical emulsifier was varied between 30 000 rpm (for smallest vesicle size) and 14 000 rpm (for largest vesicle size). Vesicles sizes below 200 nm were produced by mechanical extrusion of SUVs using filter membranes with pore sizes of 200, 100, or 50 nm. The resulting emulsion was incubated for at least 2 h at 4 °C protected from light. In order to release the dsGUVs into an aqueous buffer, the excess oil phase below the droplet layer was removed. Afterwards, PBS and 1H,1H,2H,2H‐perfluoro‐1‐octanol (PFO) were added to the droplets at a 1:1:1 volume ratio of aqueous production buffer (i.e., droplet volume): aqueous release buffer : PFO. Following 30 min of equilibration, the GUV containing aqueous top layer was transferred into microtube and PBS was added to a final volume of 2 mL. In order to purify the GUVs, a centrifugation step at >10 000 g for 15 min was performed. Finally, the GUV pellet was resuspended in 200 µL PBS.

In order to functionalize the GUVs with FasL and RANK, the total lipid concentration of the GUV solution was measured. For this, the fluorescence intensity of the integrated Atto488‐conjugated lipids was quantified using an Infinite M200 TECAN plate reader controlled by TECAN iControl software with an in‐built gain optimization and excitation/emission setting adjusted to 488/520 nm. This fluorescence intensity was then referenced to a SUV standard dilution curve of known concentration to calculate the lipid concentration of the GUV solution. Of note, this quantification method does not discriminate between different lipid‐structures (e.g., GUVs, SUVs, or micelles) that might be created as traces of contaminant side‐products during the GUV production process. Therefore, the obtained concentration values are given as total lipid and not GUV concentration values. However, it allows the authors to approximate the concentration of NTA binding sites and therefore to calculate the maximal amount of recombinant proteins that can be coupled. For coupling of FasL and RANK, the concentration NTA‐Ni^2+^‐coupled lipids was deduced from the premixed lipid ratio. The recombinant His‐tagged RANK and FasL proteins were added to the GUV solution in excess of 1:2 (protein:NTA) and incubated for 1 h at 37 °C in the dark.

### Expression of Fas‐GFP

For transfection of Fas‐GFP fusion proteins, adherent Hela cells were seeded at a density of 100 000 cells/ well in 8‐well LabTek glass‐bottom microscopy chambers in serum supplemented growth medium. Reverse transfections were performed at the time point of seeding using PolyPlus JetPrime transfection reagent. C‐terminal GFPSpark‐tagged Fas (NCBI reference sequence NM_0 07987.1) expression constructs under CMV promoter control were obtained from commercial distributer (see materials) and expressed without antibiotic selection. Transfection efficiency was observed to be ≈50%. Of note, Fas‐GFP overexpression induced considerable cell deaths even without FasL treatment.

### Dead Cell Quantification

Killing efficiency of FasL‐coupled vesicles was measured by quantification of propidium iodide (PI) staining intensity using plate reader analysis. For this, the cells were seeded at a density of 1 × 10^5^ cells/well in 96 flat‐bottom transparent well‐plates for 24 h in growth medium containing 1 µg mL^−1^ PI. Subsequently, the naive vesicles, vFasL vesicles or sFasL were added to the cells and PI fluorescence intensity was either assessed by end‐point analysis after 24 h or recorded continuously with an Infinite M200 TECAN plate reader controlled by TECAN iControl software with an in‐built gain optimization and excitation/emission setting adjusted to 535/617 nm. For continuous analysis, the plate reader was heated to 37 °C and 50 mm HEPES was added to the cell culture medium. For control measurements, cells were either left untreated (negative control) or incubated with 10% w/v Tween‐20 (positive control). PI fluorescence was measured at four different positions in each well. Three biological replicates were performed for each condition.

### Quantification of ERK Phosphorylation and Caspase‐8 Staining

For quantification of p202/204 phosphorylation, MC3T3 cells were seeded at a density of 1 × 10^5^ cells/well in 96 flat‐bottom transparent well‐plates and cultured for 24 h. Subsequently, vRANK vesicles or sRANK at a concentration of 28 ng mL^−1^ was added to the cells and incubated for 24 h. Then, the cell culture medium was removed and the cells were washed twice with 100 µL PBS and subsequently fixed by addition of 4% paraformaldehyde for 30 min. Afterwards, the cells were permeabilized by addition of 100 µL 0.1% Triton X‐100 for 10 min and washed twice with PBS for subsequent blocking with 100 µL of 1% (w/v) BSA for 30 min. Subsequently, Alexa488 conjugated anti‐ERK1/2(pT202/pY204) was added and incubated for 2 h. Excess antibody was then removed by washing twice with 1% (w/v) BSA. Cells were then stained with Hoechst33342 and imaged by epifluorescence microscopy. Fluorescence intensity of the ERK1/2(pT202/pY204) was measured by manual selection of the nuclear region (using the Hoechst33342 as counter stain) and the cytoplasm.

For analysis of caspase‐8 cleavage, Jurkat cells were seeded at a density of 100 000 cells/well in 96‐well flat bottom well plates. Subsequently, vFasL, sFasL, or naïve vesicles were added to the cells and incubated for 24 h. Then, the cell culture medium was removed and the cells were fixed by addition of 4% paraformaldehyde for 30 min. Following fixation, the cells were permeabilized by addition of 100 µL 0.1% Triton X‐100 for 10 min and washed twice with PBS for subsequent blocking with 100 µl of 1% (w/v) BSA for 30 min. Subsequently, anti‐caspase‐8 (cleaved Asp391) and 1 µg mL^−1^ Hoechst33342 was added and incubated for 2 h. Excess primary antibody was then removed by washing twice with 1% (w/v) BSA and anti‐rabbit Alexa555‐conjugated secondary antibody was added for 30 min. Wells were again washed twice with 100 µl of 1% (w/v) BSA to remove excess secondary antibody and subsequently imaged by laser scanning confocal microscopy. The same experimental procedure was applied for FADD staining using a primary anti‐human FADD antibody conjugated to AlexaFluor647.

### Fluorescence Recovery after Photobleaching (FRAP)

FRAP experiments were performed with a laser‐scanning microscope LSM 800 (Carl Zeiss AG) equipped with a 20x objective (Plan‐Apochromat 20x/0.8 M27, Carl Zeiss AG). Bleaching areas with a minimum of radius of 1 µm were selected and bleached a 100% laser intensity with 10 iterations. A minimum of 5 pre‐bleaching and 60 post‐bleaching images were acquired. Mean fluorescence intensity time profiles for the bleached area, the whole cell and background fluorescence were measured with ImageJ (NIH) software. Profile background and intensity normalization as well as data fitting, calculation of mobile fractions and calculation of *t*_half maximum_ was performed with easyFRAP web‐based tool (Zoi Lygerou, University of Patras). Diffusion coefficients were calculated following a previously published formula (*D* = 0.32*r* ^2^ *t*_1/2_^–1^).^[^ ^42^ ^]^

### Statistical Analysis

Pre‐processing of data did not include exclusion of outliers. Normalization of the data for representation purposes was performed as following: Figure 1e was normalized to the mean of the naive vesicle condition, Figure 1h was normalized to the mean of the untreated control condition, Figure 2f was normalized to the mean of the 75 nm vesicle condition, plots in Figure S2, Supporting Information, were normalized to the corresponding means of the untreated control conditions, Figure 3f was normalized to the mean of the untreated control condition, Figure 3g was normalized to the mean of the untreated control condition, and Figure S8, Supporting Information, was normalized to the mean of the untreated control condition. As stated in the Experimental section, an all plate reader‐based analysis was performed by measuring at four different positions in each well and a mean value of this was calculated. If not stated otherwise in the figure legend, all bar graphs show mean ± SD and sample sizes are given in the figure legends for the individual experiments. A minimum of three biological replicates were performed for each experiment. For statistical assessment of significant differences between the experimental conditions, one‐way analysis of variance (ANOVA) with post‐hoc testing in multiple comparison was performed. Multiple comparison using statistical hypothesis testing was performed with Bonferroni tests with a family‐wise alpha threshold and confidence level of 0.05 (95% confidence interval). *p*‐values are represented as following: >0.05 = n.s., <0.05 = *, <0.005 = **, <0.0005 = ***. All analysis were performed with Prism 9 software.

## Conflict of Interest

The authors declare no conflict of interest.

## Supporting information

Supporting Information

Supplemental Video 1

Supplemental Note 1

Supplemental Note 2

## Data Availability

The data that support the findings of this study are available from the corresponding author upon reasonable request.
