# Supplementary material for: Vesicle Induced Receptor Sequestration: Mechanisms behind Extracellular Vesicle‐Based Protein Signaling
Source: Adv Sci (Weinh). 2022 Mar 1;9(13):2200201. doi: 10.1002/advs.202200201 (PMC9069182; doi:10.1002/advs.202200201)
Supplement: Supplementary file 1 — Supporting Information [file ADVS-9-2200201-s002.pdf]

ADVANCED SCIENCE
Open Access

Supporting Information

for *Adv. Sci.*, DOI 10.1002/advs.202200201

Vesicle Induced Receptor Sequestration: Mechanisms behind Extracellular Vesicle-Based Protein Signaling

Oskar Staufer, Jochen Estebano Hernandez Bücher, Julius Fichtler, Martin Schröter, Ilia Platzman* and Joachim P. Spatz**

Supporting Information

Vesicle induced receptor sequestration: mechanisms behind extracellular vesical-based protein signaling

Oskar Staufer, Julius Fichtler, Jochen E. Hernandez Bücher, Martin Schröter, Ilia Platzman* and Joachim P. Spatz**

Supplementary table 1 Parameters for the diffusion simulation of sFasL and vFasL

variable	estimated value
charge FasL protein	$-9e$
charge sFasL protein	$-4e$
charge Fas receptor	$-2e$
relative permitivity PBS at $T = 37°C$	74.4
buffer ion density	$1.885 \cdot 10^{26}\, m^{-3}$
cut off length for Yukawa interaction potential	$0.2\, nm$
viscosity of PBS	$0.7 \cdot 10^{-3} Pa \cdot s$
temperature	$310.15\, K$
hydrodynamic radius FasL trimer	$4.7\, nm$
hydrodynamic radius sFasL trimer	$4.7\, nm$
vesicle radius	$150\, nm$
Saffman Delbrück length	$5\, \mu m$
thickness of lipid membrane	$5\, nm$
viscosity of lipid membrane ($= 2\eta_{PBS} L_{sd}/h$)	$1.40\, Pa \cdot s$
FasL diffusion constant	$0.35\, \frac{\mu m^2}{s}$
sFasL diffusion constant	$72.12\, \frac{\mu m^2}{s}$
Fas receptor diffusion constant	$0.3\, \frac{\mu m^2}{s}$
Fas receptor concentration	$12\, \mu m^{-2}$
minimum interaction distance for oligomerization	$2\, R_{FasL}$
time resolution for simulation	$10^{-3}\, s$
total number of time steps for one simulation	30000

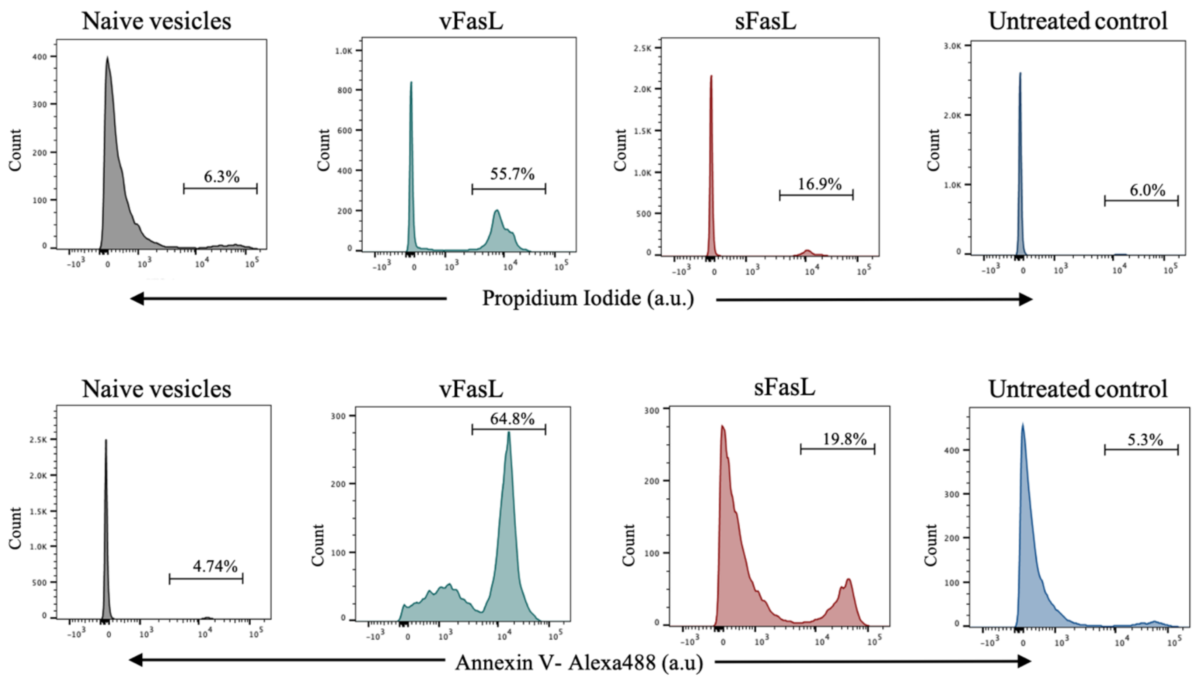

Figure S1 Correlation of PI and Annexin V-Alexa488 staining. Representative flow cytometry quantification of PI and Annexin V-Alexa488 staining in Jurkat cell cultures treated with 28 ng ml^{-1} vFasL, sFasL or naïve vesicles for 24 h. One representative example from 3 independent experiments is shown for each treatment condition.

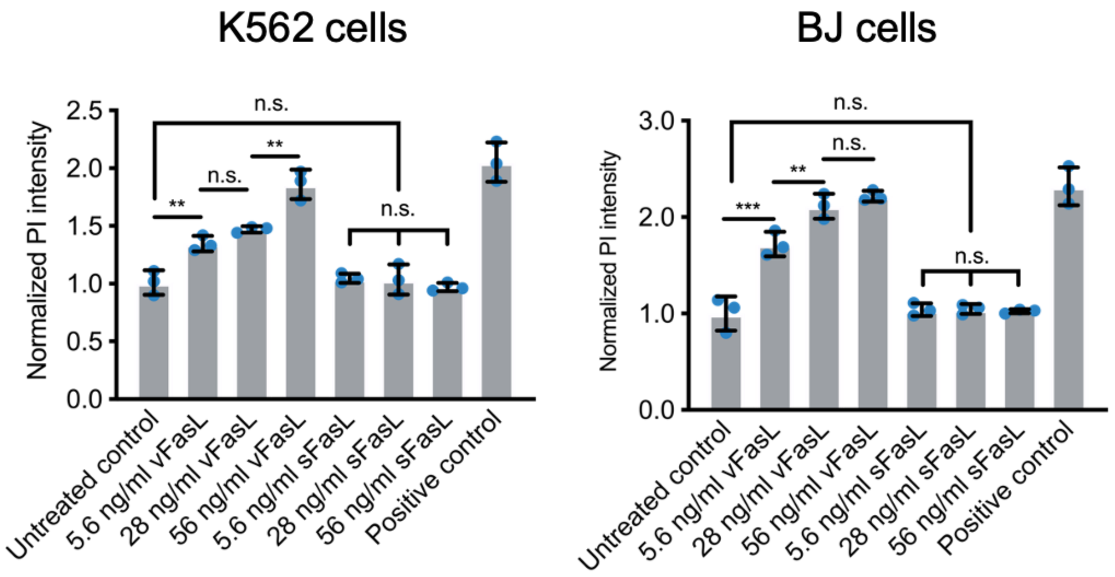

Figure S2 Comparison of apoptosis induced by vFasL and sFasL. Quantification of PI staining intensity of K562 cells (left) and BJ3 cells (right) after 24 h of incubated with varying concentrations of vFasL and sFasL. Results are shown as mean±SD from n= 3 biological triplicates. **p < 0.005, ***p < 0.0005, and n.s.= not significant with one-way ANOVA analysis and Bonferroni post-hoc testing.

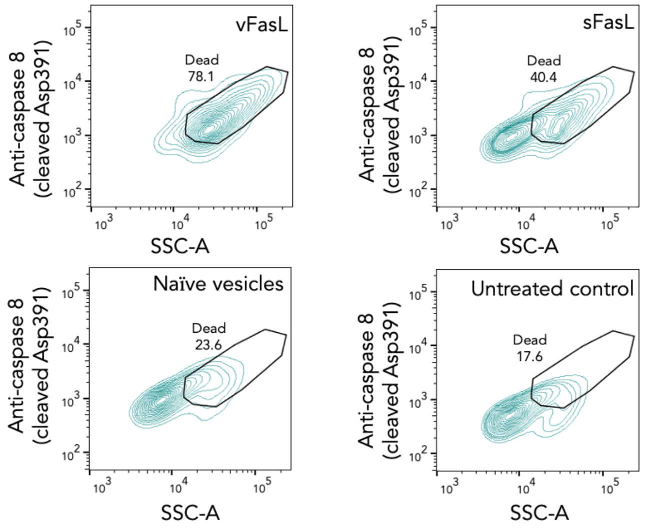

Figure S3 Flow cytometry quantification of caspase-8 cleavage. Representative flow cytometry contour plots of anti-caspase 8 (cleaved Asp391) stained Jurkat cells treated for 24 hours with vFasL, sFasL, naïve vesicles or left untreated. One representative example from 3 independent experiments is shown for each treatment condition. % of stained and dead cells is gives next to the gating settings.

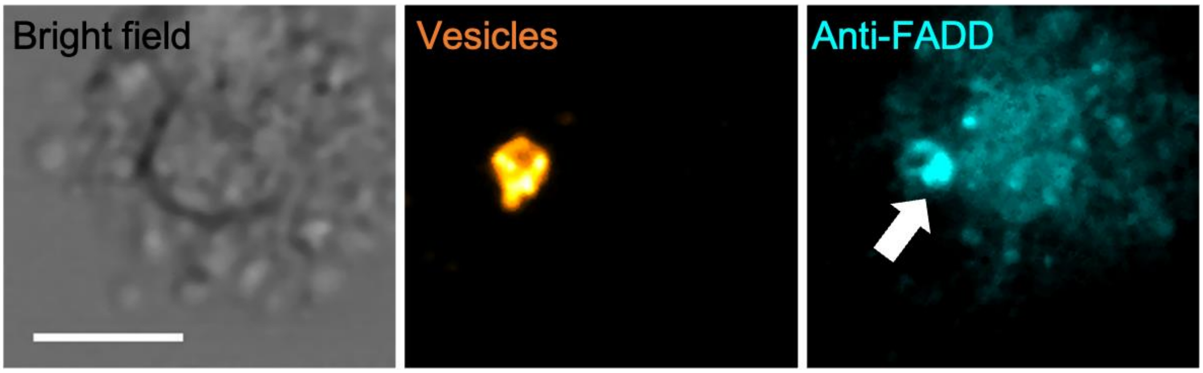

Figure S4 FADD recruitment to vesicle binding side. Representative confocal microscopy images of a Jurkat cell in attachment to a vFasL vesicle (orange) and stain with anti-human FADD AlexaFluor647 conjugate (cyan). Arrow indicates side of FADD clustering indicative for DISC formation. Scale bar is 3 µm.

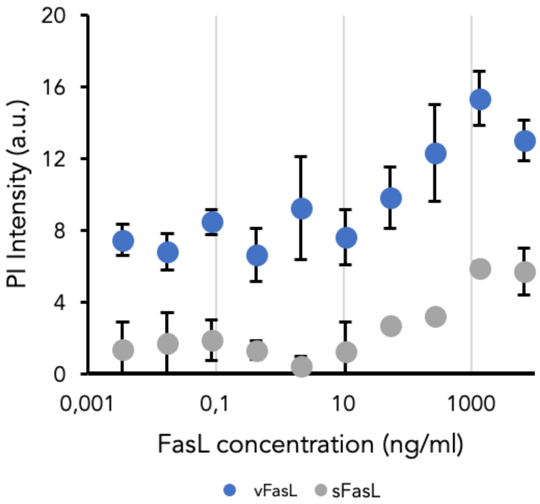

Figure S5 Dose response analysis of the pro-apoptotic potency of vFasL and sFasL. Quantification of PI staining intensity of Jurkat cells after 24 h of incubated with a dilution series of vFasL and sFasL. Results are shown as mean±SD from three biological triplicates.

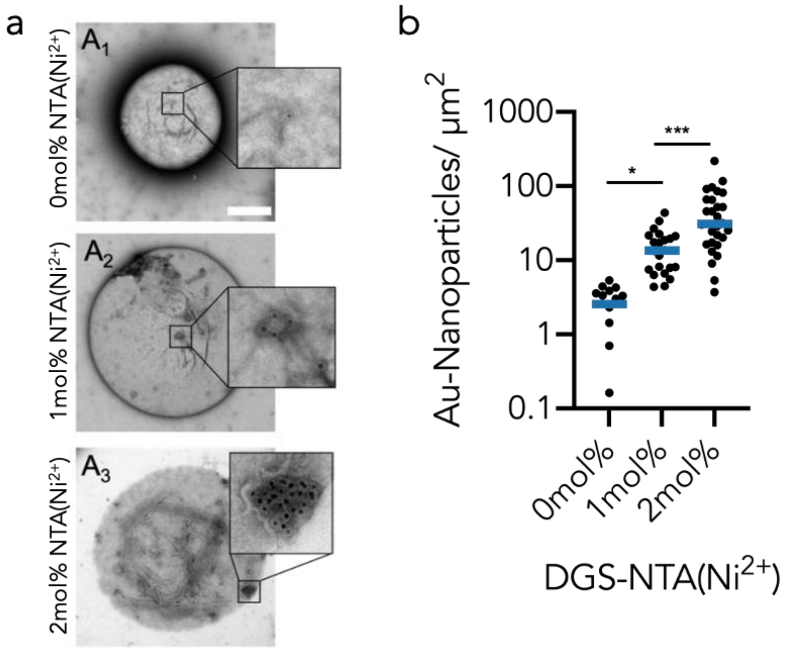

Figure S6 Assessment of NTA(Ni2+) directed protein density on vesicle membranes. (a) Representative transmission electron microscopy images of uranyl acetate negative stained vesicles harbouring varying NTA(Ni2+) concentrations and incubated with histidine-tagged protein G immobilized IgG conjugated to gold-nanoparticles. Scale bar is 500 nm. (b) Quantification of the gold nanoparticle density on the surface of vesicles harbouring different NTA(Ni2+) concentrations. Results are shown as mean±SD from n>12 single vesicles. **p < 0.005, ***p < 0.0005, and n.s.= not significant with one-way ANOVA analysis and Bonferroni post-hoc testing.

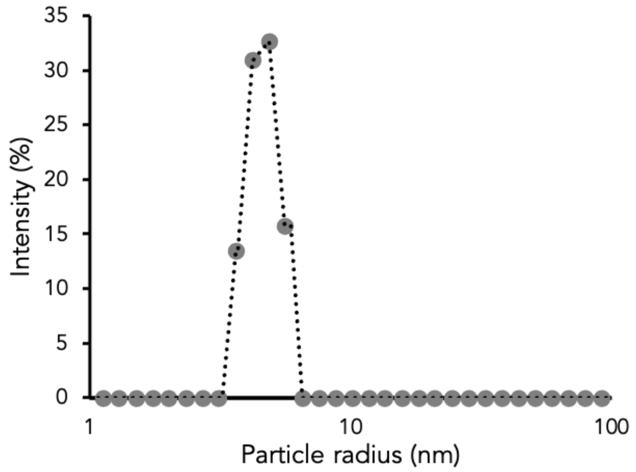

Figure S7 Analysis of FasL radius. Intensity distribution of dynamics light scattering analysis of FasL in PBS solution. Mean radius is 4.7 nm (standard deviation = ±0.7 nm).

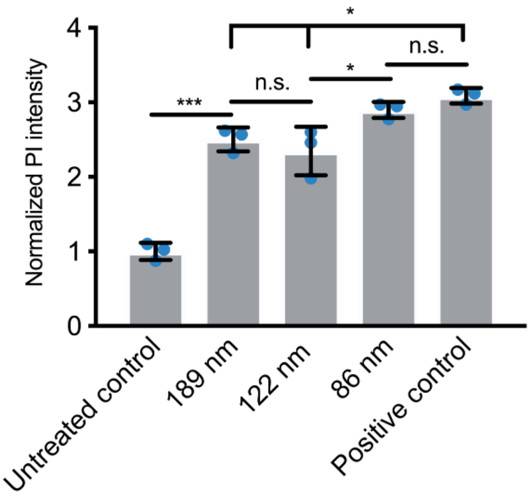

Figure S8 Quantification of bulk PI staining intensity in Jurkat cell cultures treated with vFasL vesicles of varying diameter. Results are shown as mean±SD from n= 3 biological triplicates. $*p < 0.05$, $***p < 0.0005$, and n.s.= not significant with one-way ANOVA analysis and Bonferroni post-hoc testing.

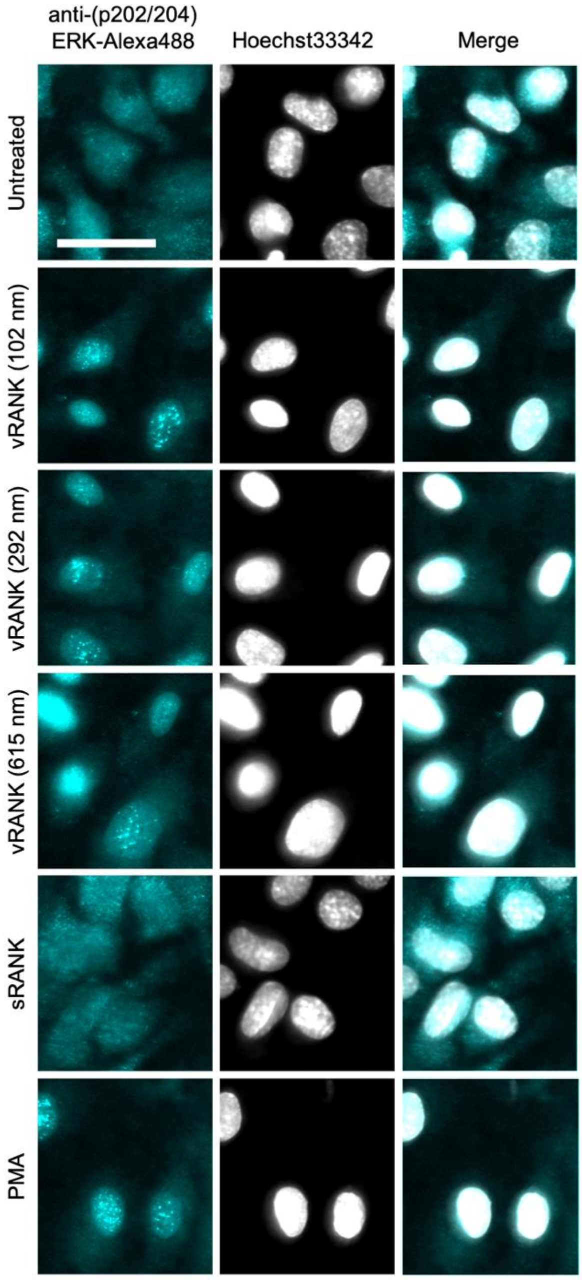

Figure S9 Nuclear translocation of ERK1/2 by sRANK and vRANK. Representative fluorescence confocal microscopy images of MC3T3 osteoblast incubated with sRANK, vRANK of varying vesicle size, PMA (positive control) or left untreated and stained with anti ERK1/2 (p202/204) (left column) and Hoechst33342 nuclear stain (right column). Right column show merged image. Scale bar is 30 µm).

Video S1 Representative fluorescence time laps analysis of a Jukrat cell incubated with fluorescent vFasL presenting vesicles (green). After contact formation, the cell rapidly undergoes apoptosis as marked by excessive blebbing and successive PI staining (red).
