# Supplementary material for: Vesicle Induced Receptor Sequestration: Mechanisms behind Extracellular Vesicle‐Based Protein Signaling
Source: Adv Sci (Weinh). 2022 Mar 1;9(13):2200201. doi: 10.1002/advs.202200201 (PMC9069182; doi:10.1002/advs.202200201)
Supplement: Supplementary file 3 — Supplemental Note 1 [file ADVS-9-2200201-s001.pdf]

Supplementary note 1

Charge-mediated formation of giant unilamellar vesicles

Giant unilamellar vesicles (GUVs) are frequently used as model membrane compartments for bottom-up assembly of protocells and synthetic cells (*1*-*3*). These spherical, usually cell-sized and water-filled lipid vesicles are enclosed by a single doubled lipid membrane which mimics the composition of biological membranes from cells and organelles. Being composed of phospholipids, these synthetic life-like mimics display central characteristics of natural membranes as found in EVs (e.g. composition, thickness or fluidity). Several techniques for the production of GUVs have been developed. For instance, (1.) electroformation of GUVs on indium tin oxide or platinum electrodes from supported lipid bilayers (*4*), (2.) inverted emulsion techniques by centrifugation, commonly referred to as “droplet transfer” methods (*5*) and (3.) octanol-assisted assembly of GUVs by dewetting using droplet-based microfluidic approaches (*6*). Each specific method comes with unique advantages and disadvantages related to throughput, flexibility in lipid composition, suitable buffers or GUV-size distribution. The work presented in this manuscript, applies droplet-stabilized and charge-mediated formation of GUVs inside water-in-oil (w/o) droplets which we described and characterized in several previous studies(*1, 3, 7, 8*) . Figure 1 of this note illustrates the key steps required for the charge-mediated formation process. Importantly, the application of this method for formation of liposomal vesicles designed to mimic natural EVs, offers a level of control over the molecular liposome composition and architecture, not-matched by any other of the described techniques.

Formation of droplet-stabilized GUVs (dsGUV) for the production of free-floating GUVS is a simple, yet flexible and high yield process which can be operated using microfluidic devices (*7*) or as a bulk-method with standard wet-lab equipment (*3*). Formation of dsGUVs is compatible with a wide variety of lipid-types and aqueous buffers with varying ion compositions. The fundamental scaffolds applied for the formation and stabilization of dsGUVs are water-in-oil (perfluorinated, bioinert oils are commonly used) droplet-emulsions stabilized by non-charged di- or triblock fluorosurfactants (*9*). These amphiphilic polymers arrange at the water-oil interface and reduce the surface tension of the droplets to form stable emulsions. The dsGUV size, and therefore the resulting GUV-size, can be adjusted by tuning the w/o droplet dimensions. In order to provide a net-charge to the droplet periphery, a second type of fluorosurfactant, carrying a deprotonated carboxyl-group (e.g. COOH-

perfluoropolyether) is added to the droplet emulsion(*7*). Furthermore, the aqueous phase used for droplet production, contains negatively charged liposomes (SUVs), suspended in a Mg^{2+} containing buffer. This solution is entrapped within the droplet lumen by microfluidic technology or mechanical emulsification. The positively charged Mg^{2+}-ions then bind to negatively charged droplet periphery and subsequently “recruit” the negatively charged liposomes to the periphery as well. In a next step, individual liposome fuse at the periphery and on the surfactant layer and form a supported lipid bilayer along the droplet boarder. As soon as complete droplet periphery is “saturated” with a lipid layer, a unilamellar vesicle is formed. Free floating GUVs are mechanically unstable. The polymer-shell support of the surfactants therefore creates a stabilizing scaffold at which complex scaffolds can be assembled. When enough SUVs reside within the droplet, the complete droplet periphery can be covered with this lipid bilayer, creating a GUV which is mechanically supported by the droplet; a droplet-stabilized GUV. When correctly adjusting the droplet diameter and total SUV concentration encapsulated into the droplets, a GUV without leftover SUVs residing in the lumen is assembled. The dsGUV formation process proceeds within seconds to minutes depending on the charges of surfactants and liposomes. All other moieties present in the initial aqueous solution (e.g. miRNA or DNA) are eventually encapsulated within the GUV lumen. After complete formation (and possible downstream modification of the dsGUVs), the vesicles can be released from their supporting shell. By this, they are extracted from a non-biological perfluorinated oil environment into an aqueous solution with physiological buffer composition (e.g. cell culture media). This release process is initiated by the addition of a destabilizing, low-molecular weight surfactant. This reduces the interfacial tension of the droplets and induces droplet destabilization, emulsion breakdown and eventually GUV release. After release, the GUVs can be further biofunctionalized with proteins, sugars or synthetic polymers (e.g. PEG). The decoration of GUVs with proteins can be easily performed by incorporating NTA(Ni^{2+}) functionalized lipids into the initial SUV mixture. In this case, His-tagged proteins can be added to the release GUVs for binding to the NTA-harbouring lipid membrane.

The most central advantages associated to the production of GUVs *via* dsGUVs are: 1. Compatibility with other microfluidic modules allwoing for sequential assembly and manipulation(*1*). 2. In contrast to electroformation, this technology allows the incorporation of a wide variety of different lipid types into the GUV membrane (e.g. positively and negatively charged lipids, saturated and poly-unsaturated lipids,

ceramides or lipids with large head groups) (*3, 7*). Additionally, no surfactant or solvent is retained in or on the released GUV. 3. DsGUV assembly can be performed with several different buffer compositions and is therefore operable with isotonic, physiological buffer enabling for subsequent incubation with cells(*3*).

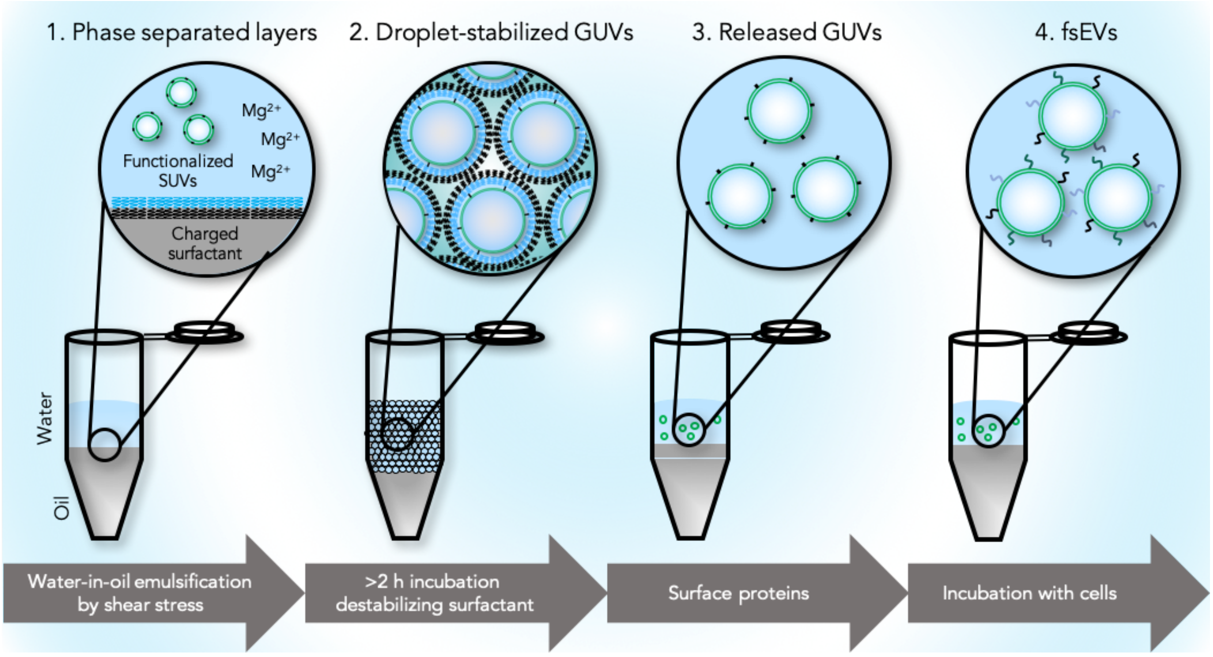

Figure 1 Schematic illustration of the charge-mediated dsGUV formation process. Negatively charged SUVs, which carry functionalized lipids (e.g. NTA(Ni^{2+}), are entrapped inside water-in-oil droplets. The aqueous phase further contains millimolar concentrations of Mg^{2+} ions. The droplet emulsion is stabilized by amphiphilic PFPE-PEG fluorosurfactants and PFPE carboxylic acid. The carboxy terminus provides a negative charge to the droplet periphery. Mg^{2+}, which are attracted to the droplet periphery by the negative charge, recruit the SUVs to the droplet periphery. Subsequently, vesicles fusion is induced and a supported lipid bilayer is created along the droplet periphery. Eventually, a dsGUV is created as the periphery is "saturated" with lipids. For release of the GUV from the stabilizing surfactant shell, a destabilizing surfactant is added, transferring the GUV into a suitable aqueous buffer. In a last step, the GUVs can be functionalized with proteins to create fully-synthetic extracellular vesicles.

1. M. Weiss *et al.*, Sequential bottom-up assembly of mechanically stabilized synthetic cells by microfluidics. *Nat Mater*, (2017).
2. K. Gopfrich, I. Platzman, J. P. Spatz, Mastering Complexity: Towards Bottom-up Construction of Multifunctional Eukaryotic Synthetic Cells. *Trends Biotechnol* **36**, 938-951 (2018).
3. K. Göpfrich *et al.*, One-pot assembly of complex giant unilamellar vesicle-based synthetic cells. *ACS Synthetic Biology*, (2019).
4. P. Meleard, L. A. Bagatolli, T. Pott, Giant unilamellar vesicle electroformation from lipid mixtures to native membranes under physiological conditions. *Methods Enzymol* **465**, 161-176 (2009).

5. A. Moga, N. Yandrapalli, R. Dimova, T. Robinson, Optimization of the Inverted Emulsion Method for High-Yield Production of Biomimetic Giant Unilamellar Vesicles. *Chembiochem* **20**, 2674-2682 (2019).
6. S. Deshpande, Y. Caspi, A. E. C. Meijering, C. Dekker, Octanol-assisted liposome assembly on chip. *Nature Communications* **7**, 10447 (2016).
7. B. Haller *et al.*, Charge-controlled microfluidic formation of lipid-based single- and multicompartment systems. *Lab Chip* **18**, 2665-2674 (2018).
8. O. Staufer, M. Schroter, I. Platzman, J. P. Spatz, Bottom-Up Assembly of Functional Intracellular Synthetic Organelles by Droplet-Based Microfluidics. *Small*, e1906424 (2020).
9. J. W. Janiesch *et al.*, Key factors for stable retention of fluorophores and labeled biomolecules in droplet-based microfluidics. *Anal Chem* **87**, 2063-2067 (2015).
10. T. Tanaka, M. Yamazaki, Membrane Fusion of Giant Unilamellar Vesicles of Neutral Phospholipid Membranes Induced by La3+. *Langmuir* **20**, 5160-5164 (2004).
