# Supplementary material for: Vesicle Induced Receptor Sequestration: Mechanisms behind Extracellular Vesicle‐Based Protein Signaling
Source: Adv Sci (Weinh). 2022 Mar 1;9(13):2200201. doi: 10.1002/advs.202200201 (PMC9069182; doi:10.1002/advs.202200201)
Supplement: Supplementary file 4 — Supplemental Note 2 [file ADVS-9-2200201-s004.pdf]

Supplementary Note 2

In this basic simulation, the 2D diffusion of FasL proteins on the surface of vesicles is compared to 3d free diffusion of soluble FasL (sFasL) proteins in an immunologic cleft using a Brownian random walk model of massless point particles with a short ranged Yukawa interaction potential. Applying the Saffman–Delbrück model to estimate diffusion coefficients of membrane bound FasL, the dynamics of ligand-induced receptor trimerization can be simulated.

1 Brownian dynamics with Langevin equation and assesment of parameters for simulation

1.1 Random walk and short ranged Yukawa potential

To describe the random walk motion of every particle in this simulation, we based our calculation on the Langevin equation

$$m \cdot \dot{v} = -\nabla U - \xi \cdot v + \sigma \cdot \eta(t) \tag{1}$$

where v is the velocity, m the mass, U the potential, $\eta(t)$ a Gaussian random noise with amplitude σ and with $\langle \eta(t) \rangle = 0$ and $\langle \eta(t)\eta(t') \rangle = 2\delta(t-t')$) every particle movement in this simulation is simulated as a random walk using the discretized (1d) version of the Langevin equation neglecting the mass:

$$x(t+\Delta t) = x(t) - D\frac{d}{dx}\left(\frac{U}{k_B T}\right)\Delta t + \sqrt{2D\Delta t} \cdot \mathcal{N}(0,1) \tag{2}$$

Here $\mathcal{N}(0,1)$ is a Gaussian distribution with vanishing mean and finite variance. The simulation was performed for a physiological buffer, which we assumed to be present in the immunological synapse. Therefore, an aqueous PBS environment was used as base formulation of the buffer. Therefore, the interaction potential U between individual proteins is modelled with a short ranged Yukawa potential [10]. Using the Debye-Hückel equation in spherical coordinates with radial distance r

$$\Delta\phi = \frac{1}{r} \cdot \frac{d^2}{dr^2}(r\phi) = \kappa^2\phi \tag{3}$$

with the Debye-Hückel screening length

$$l_{DH} = \frac{1}{\kappa} = \sqrt{\frac{\epsilon_0 \epsilon k_B T}{2e^2 n_0}} \tag{4}$$

where k_B is the Boltzmann constant, T the temperature, e the elementary charge, n_0 the particle density of the isotonic solution, ϵ_0 the vacuum permittivity and ϵ the relative permittivity. Considering a point charge Q one gets:

$$\phi(r) = \frac{Q \cdot exp(-\kappa r)}{4\pi\epsilon_0\epsilon r} \tag{5}$$

where Q is the sum of all charges of the respective proteins at pH 7.4 caclculated using the Pro Pi beta tool [6]. For human FasL, Pro134 – Leu281 of the protein UniProtKB - P48023 and for Fas UniProtKB - P25445 was used. This leads to a rough approximation of: $Q(sFasL) \approx -4e$, $Q(FasL) \approx -9e$ and $Q(Fas) \approx -2e$ (see also fig. 1).

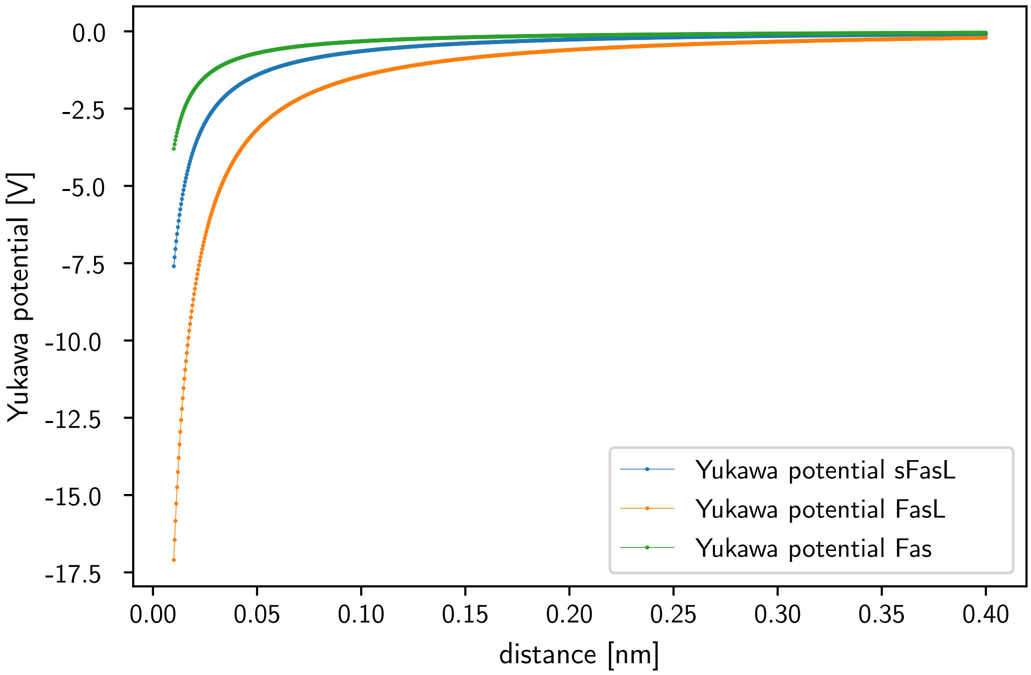

Figure 1: Short ranged Yukawa potential estimated for (s)Fas(L).

Finally, the interaction potential U between two particles for equation (2) can be estimated by multiplying the particle charge of one particle with the Yukawa potential of the other particle.

1.2 Diffusion coefficients of membrane bound Fas and (s)FasL

The diffusion coefficient of the membrane bound FasL proteins can be estimated using the hydrodynamic model of Saffman-Delbrück [5]:

$$D = \frac{k_B T}{4\pi \eta_v h} \left[ln \left(\frac{2L_{sd}}{a} \right) - \gamma \right] \tag{6}$$

Here η_v is the viscosity of the vesicle, a the radius of a columnar FasL trimer, γ the Euler–Mascheroni constant (≈ 0.577) as well as the Saffman-Delbrück length L_{sd}:

$$L_{sd} = \frac{h \cdot \eta_m}{2\eta_v} \tag{7}$$

where h is the thickness of the membrane and η_m the viscosity of the surrounding medium. Typical values for L_{sd} are in the range between $(0.1-10)\,\mu m$. Assuming $L_{sd} = 5\mu m$ and using equation (7) η_v can be estimated with $\eta_m = \eta(PBS)$. With a rough approximation of $h \approx 5nm$, the diffusion constant D_{FasL} for the Fas ligands on the vesicular membrane: $D_{FasL} \approx 0.35\,\frac{\mu m^2}{s}$. An experimentally measured value of $D_{Fas} \approx 0.3\,\frac{\mu m^2}{s}$ for the diffusion constant of membrane bounded Fas receptors was used [8]. To estimate D_{sFasL}, the Stokes-Einstein equation was used:

$$D = \frac{k_B T}{6\pi \eta R} \tag{8}$$

To determine the hydrodynamic radius R, dynamic light scattering analysis was performed. We found $R_{sFasL} = (4.7 \pm 0.7)\,\text{nm}$. As a result one gets $D_{sFasL} = (69 \pm 11)\,\frac{\mu\text{m}^2}{\text{s}}$.

2 Fundamental model of Fas hexamerization

The molecular mechanism and dynamics of Fas-FasL ligand-induced receptor trimerization are outlined in figure 2 are based on Liesche et al. (2018) [4] and Kallenberger et al. (2014) [3]. According to these previous findings, the necessary condition to induce Fas-based apoptotic signaling is the spatial proximity of two ligand-receptor trimer complexes.

With the assumption that (s)FasL primarily exists in it's trimerized form [4], every triple-ligand eventually needs to bind three receptors. This can be achieved by gradually binding of three single Fas receptors as well as a combination of a dimerized receptor and a single receptor to a ligand trimer (fig. 2). In this context Liesche et al. reported that at a density of less than ten receptors per micrometer, Fas receptors exist at 58%

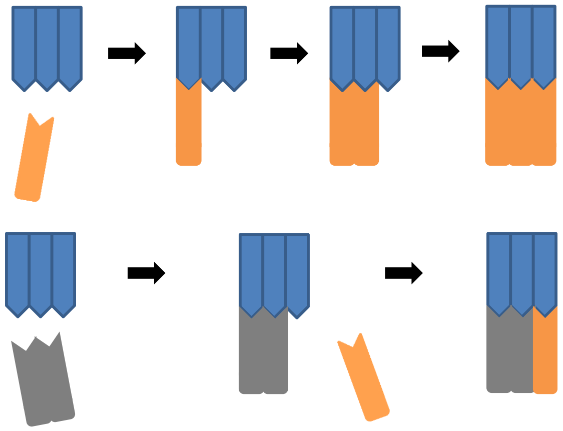

Figure 2: Basic mechanisms of trimerization of (s)FasL-receptor complexes. (s)FasL (blue), single Fas receptor (orange), Fas receptor dimer (grey).

in a monomeric and 42% in a dimeric form [4]. The major output of the simulation is the number of hexamerization events in a given time window, which we assume to be a correlating measure for the apoptotic signaling. For this, the number of interactions of Fas-FasL trimers, which we defined as the spatial proximity of less than two times the hydrodynamic radius of two ligand-receptor trimer complexes, are counted.

3 sFasL apoptosis counting

First, the free diffusion of sFasL in three dimensions in an immunologic synapse of size $(2000 \times 2000 \times 2000)$ nm^3 with a continuous uniform distribution for initialisation is analysed, where sFasL diffuses to the target membrane with Fas receptors . The cubic immunologic synapse was implemented as an infinite boxing model with the target receptor membrane at the bottom of this box and sFasL is found in its trimeric state because of the nM affinity of single sFasL proteins to each other (Daburon et al. (2013)). The receptors are therefore only able to move in the membrane plane, wherefore as soon as a soluble ligand has bound to a receptor forming a ligand-receptor complex, its (lateral) movement is restricted to a two-dimensional plane. After binding, we assumed that the Fas-FasL complex can than to some extend diffuse out of the immunological synappse, as the plane of the target membrane is much larger than the immunologic synapse. According to Liesche et al. [4], a receptor density of $c_{rec} = 12\ receptors/\mu m^2$ was assumed. For a total surface area of the target membrane of approximately 1000 μm^2, this results in 12000 receptors would need to be simulated. As with conventional computer resources, it

is impossible to simulate this in a realistic period of time, a dynamic time-dependant area size of the target membrane with receptors was implemented. Using the mean squared displacement (MSD) of a random walk, the quadratic plane size of the target membrane can be chosen in a way that only receptors, which are able to move on average to the cubic immunologic cleft with edge length a during simulation time t, are taken into account. As a result the total number n_{rec} of receptors for each simulation is:

$$n_{rec} = c_{rec} \cdot (a + 2 \cdot \sqrt{2Dt})^2 \tag{9}$$

Using $t = 30s$, $n_{rec} = 1319$ is obtained.

4 VFasL apoptosis counting

In order to simulate the vFasL induced Fas-hexamerization, the complex geometry of vesicles attaching to the target cell membrane and finally sustaining the FasL-Fas interactions on this membrane, was modelled. Towards this direction, the 2d movement of ligands bound on the spherical surface of a vesicle with radius R_{ves} was projected to an infinite quadratic plane with edge length:

$$a = \sqrt{4\pi \cdot R_{ves}^2} \approx 3.545 \cdot R_{ves} \tag{10}$$

This squared plane was now set into the much larger receptor-target membrane. For possible Fas-FasL interactions, it is important not to take the whole ligand plane into account, as in fact not the whole vesicle surface is able to attach to the target cell membrane. In order to estimate this area an electron tomography image of vesicles released into the immunological synapse [2] was analysed using *ImageJ*. Few obtained for the edge length a_{int} of the interaction plane localised within the squared vesicle plane ain't is obtained:

$$a_{int} \approx 0.6978 \cdot R_{ves} \tag{11}$$

Finally, in this simulation a total of three simulation planes are modelled:

- dynamic receptor plane of size $(a + 2 \cdot \sqrt{2Dt})^2$ according to equation 9
- projected total vesicle plane of size a^2 within the receptor plane
- reduced vesicle plane of size a_{int}^2 within the projected total vesicle plane. Only in this plane, ligand receptor interactions are taken into account

Moreover, in contrast to the soluble model, vesicles need a certain period of time in order to move to the receptor membrane until the described interaction kinetics can start. For this reason an additional simulation of only one vesicle in the immunologic cleft

moving to the target membrane was performed. The initial position for every simulation was a random uniform distribution in the x-y-plane parallel to the target membrane with a height in z-direction of $2000nm$. Finally, after a loop of 1000000 simulations the average time for one vesicle to diffuse to the target cell membrane can be estimated to:

$$t_{ves} = (0.94 \pm 0.04)\,\mathrm{s} \tag{12}$$

References

[1] Whole simulation with user friendly program environment: simply run the *main_ (s)FasL_ user.ipynb* file with a python Jupyter notebook . URL: linkwillbeadded.

[2] K Choudhuri et al. *Polarized release of TCR-enriched microvesicles at the center of the T cell immunological synapse.* 2013.

[3] Stefan Matthias Kallenberger. "Variability in cellular signal transduction networks". PhD thesis. 2014, pp. 63–69.

[4] Clarissa Liesche et al. "CD95 receptor activation by ligand-induced trimerization is independent of its partial pre-ligand assembly". In: *bioRxiv* (2018), p. 293530.

[5] PG Saffman and M Delbrück. "Brownian motion in biological membranes". In: *Proceedings of the National Academy of Sciences* 72.8 (1975), pp. 3111–3113.

[6] *UniProt.* last downloaded 2019-06-09. URL: https://www.protpi.ch/Calculator/ProteinTool#Results.

[7] *UniProt.* last downloaded 2019-07-15. URL: https://www.uniprot.org/uniprot/P48023.

[8] Arun S Varadhachary et al. "Phosphatidylinositol 3-kinase blocks CD95 aggregation and caspase-8 cleavage at the death-inducing signaling complex by modulating lateral diffusion of CD95". In: *The Journal of Immunology* 166.11 (2001), pp. 6564–6569.

[9] Harald Wajant. "Principles and mechanisms of CD95 activation". In: *Biological chemistry* 395.12 (2014), pp. 1401–1416.

[10] Hideki Yukawa. "On the interaction of elementary particles. I". In: *Proceedings of the Physico-Mathematical Society of Japan. 3rd Series* 17 (1935), pp. 48–57.

Appendix

variable	estimated value
charge FasL protein	$-9e$
charge sFasL protein	$-4e$
charge Fas receptor	$-2e$
relative permitivity PBS at $T = 37°C$	74.4
buffer ion density	$1.885 \cdot 10^{26}\, m^{-3}$
cut off length for Yukawa interaction potential	$0.2\, nm$
viscosity of PBS	$0.7 \cdot 10^{-3} Pa \cdot s$
temperature	$310.15\, K$
hydrodynamic radius FasL trimer	$4.7\, nm$
hydrodynamic radius sFasL trimer	$4.7\, nm$
vesicle radius	$150\, nm$
Saffman Delbrück length	$5\, \mu m$
thickness of lipid membrane	$5\, nm$
viscosity of lipid membrane ($= 2\eta_{PBS} L_{sd}/h$)	$1.40\, Pa \cdot s$
FasL diffusion constant	$0.35\, \frac{\mu m^2}{s}$
sFasL diffusion constant	$72.12\, \frac{\mu m^2}{s}$
Fas receptor diffusion constant	$0.3\, \frac{\mu m^2}{s}$
Fas receptor concentration	$12\, \mu m^{-2}$
minimum interaction distance for oligomerization	$2\, R_{FasL}$
time resolution for simulation	$10^{-3}\, s$
total number of time steps for one simulation	30000

Table 1: Variables and estimated values used for the simulation.
